# (−)-Epigallocatechin gallate inhibits stemness and tumourigenicity stimulated by AXL receptor tyrosine kinase in human lung cancer cells

**DOI:** 10.1038/s41598-020-59281-z

**Published:** 2020-02-12

**Authors:** Kozue Namiki, Pattama Wongsirisin, Shota Yokoyama, Motoi Sato, Anchalee Rawangkan, Ryo Sakai, Keisuke Iida, Masami Suganuma

**Affiliations:** 1 Graduate School of Science and Engineering, Saitama University, Saitama, 338-8570 Japan; 2 Research Institute for Clinical Oncology, Saitama Cancer Center, Saitama, 362-0806 Japan; 3 School of Medical Science, University of Phayao, Phayao, Thailand 56000; 4 Molecular Chirality Research Center and Department of Chemistry, Graduate School of Science, Chiba University, Chiba, 263-8522 Japan

**Keywords:** Cancer prevention, Cancer stem cells

## Abstract

Cancer stem cells (H1299-sdCSCs) were obtained from tumour spheres of H1299 human lung cancer cells. We studied low stiffness, a unique biophysical property of cancer cells, in H1299-sdCSCs and parental H1299. Atomic force microscopy revealed an average Young’s modulus value of 1.52 kPa for H1299-sdCSCs, which showed low stiffness compared with that of H1299 cells, with a Young’s modulus value of 2.24 kPa. (−)-Epigallocatechin gallate (EGCG) reversed the average Young’s modulus value of H1299-sdCSCs to that of H1299 cells. EGCG treatment inhibited tumour sphere formation and *ALDH1A1* and *SNAI2 (Slug)* expression. AXL receptor tyrosine kinase is highly expressed in H1299-sdCSCs and *AXL* knockdown with siAXLs significantly reduced tumour sphere formation and *ALDH1A1* and *SNAI2 (Slug)* expression. An AXL-high population of H1299-sdCSCs was similarly reduced by treatment with EGCG and siAXLs. Transplantation of an AXL-high clone isolated from H1299 cells into SCID/Beige mice induced faster development of bigger tumour than bulk H1299 cells, whereas transplantation of the AXL-low clone yielded no tumours. Oral administration of EGCG and green tea extract (GTE) inhibited tumour growth in mice and reduced p-AXL, ALDH1A1, and SLUG in tumours. Thus, EGCG inhibits the stemness and tumourigenicity of human lung cancer cells by inhibiting AXL.

## Introduction

Green tea and (−)-epigallocatechin gallate (EGCG), the main constituent of green tea catechins, prevent cancer in humans, as demonstrated in phase II clinical trials, which showed that EGCG prevented colorectal adenoma recurrence and prostate cancer development from high-grade prostate intraepithelial neoplasia^1–4^. Numerous investigators have reported therapeutic effects in various human cancer cell lines by combining EGCG and other green tea catechins with anticancer compounds, including anticancer drugs, nonsteroidal anti-inflammatory drugs, and phytochemicals^5,6^. Because EGCG inhibits the expression of stemness marker genes and epithelial–mesenchymal transition (EMT)-related genes in human cancer stem cells (CSCs) of the breast, lung, prostate and liver, CSCs are targets of EGCG for cancer prevention and therapy^7^.

Using atomic force microscopy (AFM), Gimzewski’s laboratory first reported that metastatic cancer cells obtained from the pleural fluids of various cancer patients showed lower average Young’s modulus values, indicating lower cell stiffness, than those of normal mesothelial cells from pleural effusion^8^. Furthermore, they showed that treatment with green tea extract (GTE) increased the average Young’s modulus values for metastatic cancer cells (i.e., reversed the values to those of normal cell stiffness levels)^9^. Our previous study revealed that EGCG increased the stiffness of H1299 and Lu99 human non-small cell lung cancer (NSCLC) cells, inhibited the high expression of EMT-related proteins, such as vimentin and SLUG, and reduced cell motility^10^. To investigate the inhibitory effects of EGCG on the biophysical properties of CSCs, we enriched CSCs from the tumour spheres of H1299 cells (H1299-sdCSCs). H1299-sdCSCs overexpressed both stemness marker genes (*CD133, ALDH1A1, NANOG, SOX2*, and *OCT4*) and EMT-related genes (*CDH2 (N-cadherin), VIM (Vimentin), SNAI1(Snail), SNAI2 (Slug)*, and *ZEB1*). We aimed to characterize the critical factors regulating the biophysical and stemness properties of H1299-sdCSCs and identify targets for preventing cancer with EGCG.

We recently reported that AXL receptor tyrosine kinase (AXL) stimulates cell softening and motility in H1299 and Lu99 human lung cancer cells^11^. *AXL* was isolated as a novel transforming gene from patients with chronic myelogenous leukemia; the name ‘*AXL*’ was derived from the Greek word for ‘non-controlled’^12,13^. The AXL protein belongs to the TAM (TYRO3, AXL, and MER) receptor tyrosine kinase family and is activated by growth arrest-specific 6 (GAS6), resulting in autophosphorylation of the Y702 and Y703 residues^14^. High coexpression of AXL and GAS6 is a risk factor for poor survival in lung cancer patients^15^. AXL has attracted attention as a driver of therapeutic resistance, i.e., high AXL expression is closely associated with the acquisition of drug resistance in lung and breast cancer cells^16,17^. Furthermore, AXL stimulates the self-renewal of stem cells from breast cancer, chronic myelogenous leukaemia, and glioblastoma^18–20^. Our experiments showed that a human NSCLC cell line, H1299, expressed high amounts of both AXL and phosphorylated AXL (p-AXL, an active form) and revealed that treating H1299 cells with *AXL*-targeted siRNA increased the average Young’s modulus value. These findings indicated that the stiffness of the H1299 cells was reversed and that motility was inhibited to the levels of the low-motile high-stiffness H1703 NSCLC cell line, as if the cells had been treated with EGCG^11^. Thus, AXL appears to play a significant role in the stemness of H1299-sdCSCs.

We revealed that H1299-sdCSCs possessed less stiffness and softer elasticity than the H1299-parental cells, and EGCG reversed the lower stiffness of the H1299-sdCSCs to the same stiffness of the H1299-parental cells, suggesting that AXL expression was inhibited in the H1299-sdCSCs. Treatment with EGCG and siAXL similarly induced the following inhibitory effects on the H1299-sdCSCs: 1) a reduction in tumour sphere formation; 2) inhibition of *ALDH1A1* and *SNAI2 (Slug)* but not other stemness markers or EMT-related genes; and 3) a reduction in aldehyde dehydrogenase (ALDH)-positive cells. Furthermore, high AXL and p-AXL levels in the AXL-high clone strongly induced the tumourigenicity of H1299 cells in SCID/Beige mice, and oral administration of EGCG + GTE inhibited the tumour growth of H1299 cells by reducing p-AXL, SLUG and ALDH1A1 levels. The results suggest that AXL contributes to the stemness of H1299-sdCSCs and is a unique target for cancer prevention with EGCG.

## Results

### Inhibitory effects of green tea catechins on the tumour sphere formation of H1299 cells

We first confirmed that H1299 human lung cancer cells produced 35.0 ± 8.7 tumour spheres under non-attached and serum-free culture conditions. To determine the stemness properties, tumour spheres were first separated from parental H1299 cells using a filter with a pore size of more than 77 µm. Since the cells of the tumour spheres differed qualitatively from the parental H1299 cells, we named the cells H1299-sdCSC. H1299-sdCSCs had significantly increased expression of the stemness marker genes, *CD133* (2.2-fold), *ALDH1A1* (69.9-fold), *NANOG* (8.5-fold), *SOX2* (19.2-fold), and *OCT4* (5.0-fold), and the EMT-related genes, *CDH2 (N-cadherin)* (6.0-fold), *VIM (Vimentin)* (1.4-fold), *SNAI (Snail)* (4.7-fold), *SNAI2 (Slug)* (19.3-fold), and *ZEB1* (6.4-fold), compared with the parental H1299 (Supplementary Fig. S1).

**Figure 1:**
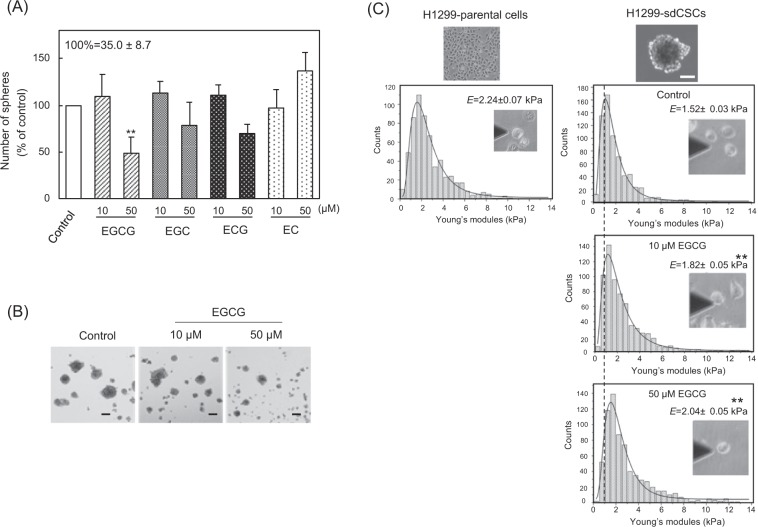
EGCG inhibited tumour sphere formation and increased stiffness of H1299-sdCSCs. (**A**) H1299 cells were cultured in serum-free medium in the presence of 10 µM or 50 µM green tea catechins, including EGCG, EGC, ECG, and EC, for 3 weeks. The results are expressed as the mean % of the control ± SD from three independent experiments. (**B**) Representative images of tumour spheres treated with vehicle and EGCG. Black bars indicate 100 µm. (**C**) Histogram of average Young’s modulus values for cells separated from H1299-sdCSCs compared with those from H1299-parental cells. Separated cells from H1299-sdCSCs were attached to a BAM-coated dish, then measured via AFM. The black line indicates the lognormal fitting curve for each treatment, and the dotted line indicates the average Young’s modulus value of the control H1299-sdCSCs. *E* values indicate the average Young’s modulus value ± SD from three independent experiments. **P < 0.01, *P < 0.05.

EGCG induces anticancer activity by inhibiting the expression of stemness marker genes and self-renewal of human CSCs^7^. Green tea contains at least four green tea catechins, which are divided into two groups: the cancer preventive group, which includes EGCG, (−)-epigallocatechin (EGC), and (−)-epicatechin gallate (ECG); and the inactive group, which includes (−)-epicatechin (EC)^21^. The inhibitory effects of green tea catechins on H1299-sdCSCs were investigated in a tumour sphere formation assay, using two concentrations (10 and 50 µM) of each catechin. Treatment with 50 µM EGCG, 50 µM EGC, and 50 µM ECG reduced the numbers of tumour spheres from 35.0 ± 8.7 to 16.0 ± 3.5, 27.6 ± 5.5, and 23.7 ± 2.1, respectively. Compared with the control, tumour sphere formation was reduced by 51.9%, 21.1%, and 30.7%, respectively. EC did not reduce tumour sphere formation (Fig. 1A). Among the catechins, EGCG inhibited tumour sphere formation most effectively, and previous results support that EGCG is the most active cancer preventive compound^4^. Tumour spheres treated with EGCG were smaller than non-treated cells (Fig. 1B). Importantly, EGCG significantly inhibited the proliferation of H1299-sdCSCs. The effects of EGCG and EC were also confirmed in another NSCLC cell line, Lu99, as shown in Supplementary Fig. S2.

### Biophysical properties of H1299-sdCSCs and their inhibition by EGCG

We previously reported the results obtained by AFM that the highly metastatic mouse melanoma variant, B16-F10, had the lowest average Young’s modulus value among all melanoma variants tested^21,22^. Thus, we believe that cell stiffness, a biophysical phenotype of cancer, is a suitable indicator of metastatic potential. To measure cell stiffness, the trypsinized H1299-sdCSCs and H1299 cells were attached to biocompatible anchor for membrane (BAM)-coated dishes^23^. AFM measurement revealed that the average Young’s modulus values were 2.24 ± 0.07 kPa for H1299 and 1.52 ± 0.03 kPa for H1299-sdCSCs (Fig. 1C). This indicated that H1299-sdCSCs possessed less stiffness and softer elasticity than H1299. Furthermore, treatment with 10 µM and 50 µM EGCG increased the average Young’s modulus values of H1299-sdCSCs from 1.52 ± 0.03 kPa to 1.82 ± 0.05 kPa and 2.04 ± 0.05 kPa, respectively (Fig. 1C). During AFM measurement, we observed that EGCG-treated H1299-sdCSCs showed a similar adherent phenotype to H1299-parental, and that suspended H1299 cells quickly adhered to BAM-coated dishes, but H1299-sdCSCs did not. We assumed that EGCG reversed the biophysical property of H1299-sdCSCs to that of H1299-parental cells, which was supported by our previous results that EGCG increased the stiffness of adhered cells in parental H1299^10^.

### Inhibition of *ALDH1A1* and *SNAI2 (Slug)* expression in H1299-sdCSCs treated with EGCG was associated with reduced ALDH-positive cells

**Figure 2:**
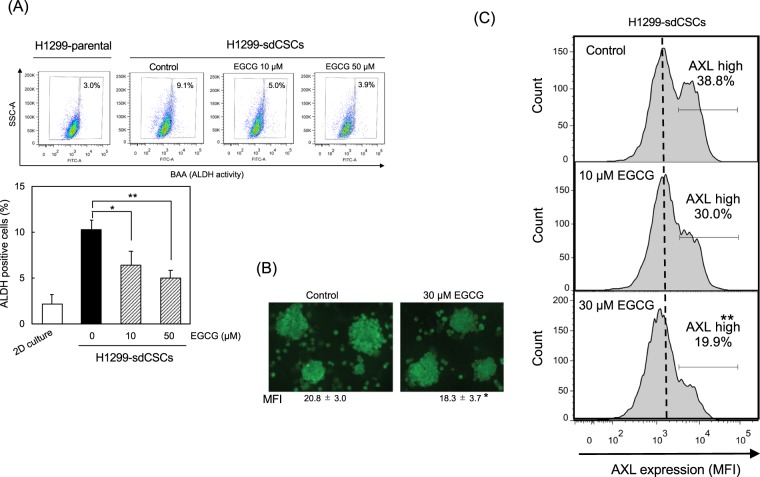
Reduced ALDH-positive cells, ALDH levels, and AXL levels in H1299-sdCSCs. (**A**) Average percentage of ALDH-positive cells in H1299-sdCSCs. The upper figure shows representative results detected by Aldefluor flow cytometry assay in H1299-parental cells and H1299-sdCSCs treated with vehicle (Control), 10 µM, and 50 µM EGCG. Baseline fluorescence was established by inhibiting ALDH activity with DEAB. The lower graph shows the mean ± SD of the percentage of ALDH-positive cells from three independent experiments. (**B**) Representative images of immunocytochemical staining of ALDH1A1 in non-treated and 30 µM EGCG-treated H1299-sdCSCs. Numbers indicate the mean fluorescence intensity (MFI) of 15 tumour spheres from three independent experiments. (**C**) AXL protein levels in the H1299-sdCSCs were examined via flow cytometry using anti-AXL antibody and AlexaFluor 647-labeled secondary antibody. The dotted line indicates MFI in the control H1299-sdCSCs. Three independent experiments were conducted. **P < 0.01, *P < 0.05.

The inhibitory effects of EGCG on the expression of five stemness marker genes and five EMT-related genes in H1299-sdCSCs were studied. Treatment with 10 µM EGCG reduced *ALDH1A1* expression to 0.23 ± 0.12, and treatment with 50 µM EGCG reduced *SNAI2 (Slug)* expression to 0.20 ± 0.12 compared with that of H1299-sdCSCs without EGCG (Table 1). However, EGCG did not change the expression of other stemness marker genes (*CD133, NANOG, SOX2*, and *OCT4*) or other EMT-related genes (*CDH2 (N-cadherin), VIM (vimentin), SNAI1(Snail)*, and *ZEB1* (Table 1). Other investigators similarly reported that ALDH is highly expressed in spheroid-derived CSCs of human colorectal cancer (HCT116-SDCSCs) compared with that in parental cells, and that ALDH activity is associated with *ALDH1A1* expression and high tumourigenicity^24,25^. The percentage of ALDH-positive cells in both H1299 and H1299-sdCSCs was determined by Aldefluor flow cytometry, and we found that the proportion of ALDH-positive cells in H1299-sdCSCs were approximately 3- to 5-fold more than that in H1299 cells (Fig. 2A). In addition, treatment with 10 µM and 50 µM EGCG dose-dependently decreased the percentages of ALDH-positive cells in H1299-sdCSCs by approximately 0.6- and 0.4-fold, respectively (Fig. 2A). We confirmed that EGCG reduced ALDH1A1 protein levels in H1299-sdCSCs by immunocytochemical analysis (Fig. 2B). The results suggest that ALDH and SLUG in H1299-sdCSCs contributed to stemness, which was inhibited by EGCG.

**Table 1:** Specific inhibition of *ALDH1A1* and *SNAI2* (*Slug)* gene expressions in H1299-sdCSCs after treatment with EGCG and siAXL.

	Relative expression (fold of the control)†				
	EGCG (10 μM)	EGCG (50 μM)	siControl	siAXL-1	siAXL-2
**Stemness genes**					
*CD133*	1.07 ± 0.33	1.44 ± 0.01	1.64 ± 0.69	5.64 ± 0.83	1.70 ± 0.46
*Nanog*	1.05 ± 0.47	1.11 ± 0.47	1.14 ± 0.65	2.30 ± 1.24	1.35 ± 0.60
*Sox2*	0.70 ± 0.32	0.96 ± 0.08	1.32 ± 0.37	1.42 ± 0.65	2.08 ± 1.09
*Oct4*	1.01 ± 0.56	1.11 ± 0.39	1.12 ± 0.37	1.92 ± 1.16	1.25 ± 0.52
*ALDH1A1*	0.23 ± 0.12*	1.53 ± 1.32	1.23 ± 0.41	0.78 ± 0.87	0.31 ± 0.20*
**EMT-marker genes**					
*CDH2 (N-Cadherin)*	1.26 ± 1.08	0.99 ± 0.46	2.23 ± 0.06	2.33 ± 0.89	2.03 ± 1.13
*VIM (Vimentin)*	1.00 ± 0.36	1.09 ± 0.27	1.07 ± 0.22	1.00 ± 0.04	1.03 ± 0.26
*SNAI1(Snail)*	1.14 ± 0.24	1.47 ± 0.29	1.05 ± 0.09	0.90 ± 0.41	0.87 ± 0.23
*SNAI2 (Slug)*	0.68 ± 0.18	0.20 ± 0.12*	1.42 ± 0.58	0.41 ± 0.24**	0.38 ± 0.19**
*ZEB1*	1.24 ± 0.25	1.09 ± 0.09	1.32 ± 0.21	1.25 ± 0.14	1.25 ± 0.30

^†^Expression level of each gene normalized by GAPDH in control H1299-sdCSCs expressed as 1.0.

*P < 0.05, **P < 0.01.

### EGCG inhibited AXL expression in H1299-sdCSCs

AXL is a receptor tyrosine kinase. We previously reported that AXL stimulates H1299 cell softening, and treatment with the R428 AXL inhibitor reversed it^11^. As aforementioned, H1299 cells contain both high amounts of AXL protein and p-AXL at Y702. H1299 treated with 50 µM EGCG and 50 µM EGC had reduced p-AXL/AXL ratios of 0.58 and 0.64, respectively (Supplementary Fig. S3), suggesting that green tea catechins inhibited AXL activation.

We next studied the relationship between stemness and AXL protein expression in EGCG-treated H1299sd-CSCs. Flow cytometric analysis using anti-AXL antibody and AF647-labeled secondary antibody revealed that H1299-sdCSCs consisted of two populations with different AXL protein expression levels on the membrane: 38.8% for the high AXL-expressing population (AXL-high) and 61.2% for the low AXL-expressing population (AXL-low) (Fig. 2C). Treatment with 10 µM and 30 µM EGCG reduced the percentage of the AXL-high population from 38.8% to 30.0% and 19.9%, respectively, and decreased the mean fluorescence intensity (MFI) of the AXL protein from 2,199 ± 100 to 1,655 ± 32 and 1,585 ± 277 (Fig. 2C), respectively, indicating that EGCG inhibited AXL protein expression in H1299-sdCSCs.

### Contribution of AXL to H1299-sdCSC stemness

**Figure 3:**
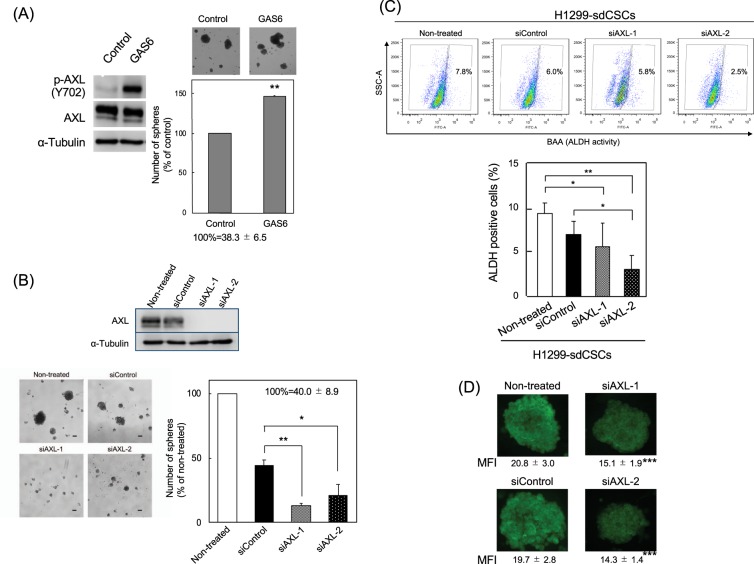
Involvement of AXL in tumour sphere formation and ALDH activity in H1299-sdCSCs. (**A**) Treatment of H1299-sdCSCs with GAS6 (250 ng/ml) significantly increased phosphorylated AXL (p-AXL Y702) (p-AXL/AXL) compared with that in control cells, as analysed by western blotting. α-Tubulin was used as a control. This blot is a part of Supplementary Fig. S2B. Tumour sphere formation was stimulated by treatment with GAS6 (100 ng/ml) for 3 weeks. The number of tumour spheres is shown as a percentage of the control. Three independent experiments were conducted. (**B**) Knockdown of *AXL* with siAXL-1 and siAXL-2 inhibited tumour sphere formation. Cells were treated with siAXLs or siControl for 2 days and then cultured in serum-free medium for 3 weeks. Images show representative tumour spheres. Percentages of tumour spheres of non-treated cells are shown in the graph. The results are the mean ± SD of three independent experiments. (**C**) Knockdown of *AXL* with siAXL-1 and siAXL-2 reduced the percentage of ALDH-positive cells. ALDH-positive cells in H1299-sdCSCs treated with siControl, siAXL-1, or siAXL-2 were detected by Aldefluor flow cytometry assay. The upper figure shows the representative results. The graph shows the average percentage of ALDH-positive cells in three independent experiments. Full-length blots/gels are presented in Supplementary Information WB-1 and -2. (**D**) Representative images of ALDH1A1 immunocytochemical staining in non-treated, siControl-, siAXL-1-, and siAXL-2-treated H1299-sdCSCs. Numbers indicate MFI of 15 tumour spheres from three independent experiments. *P < 0.05, **P < 0.01, ***P < 0.001.

GAS6, a protein related to the anticoagulation factor protein S, is a ligand for the AXL and TAM family of receptor tyrosine kinases^14^. To study the contribution of AXL activation to H1299-sdCSC stemness, the effect of GAS6 on tumour sphere formation was examined. Treatment of H1299 cells with GAS6 at 250 ng/ml increased p-AXL protein levels by approximately 8-fold, suggesting that GAS6 induced AXL activation (Fig. 3A). GAS6 treatment in the tumour sphere assay induced significantly larger spheres than those from cells without GAS6 and increased the number of tumour spheres from 100% (38.3 ± 6.5) to 146% (56.0 ± 9.5; Fig. 3A). Importantly, activating AXL with GAS6 stimulated tumour sphere formation and stemness.

We next transfected siAXL-1 and siAXL-2 into H1299 cells to reduce AXL protein levels (Fig. 3B). Treatment with siAXL-1 and siAXL-2 reduced the numbers of tumour spheres to 13.3% (5.3 ± 1.5) and 20.8% (8.3 ± 3.5) in the non-treated H1299 (40.0 ± 8.9), respectively, and the inhibitory effect with siAXLs was more effective than 44.3% (17.7 ± 4.0) with the siControl (Fig. 3B). Consistent with evidence showing that EGCG reduces *ALDH1A1* and *SNAI2 (Slug)* expression, treatment with siAXL-1 and siAXL-2 also reduced *ALDH1A1* (0.78 and 0.31) and *SNAI2 (Slug)* (0.41 and 0.38) expression among five stemness marker genes and five EMT-related genes in H1299-sdCSCs (Table 1). Furthermore, H1299-sdCSCs treated with two siAXLs had a reduced percentage of ALDH-positive cells, as detected by flow cytometry, and reduced ALDH1A1 protein levels, as determined by immunocytochemistry, compared with non-treated H1299-sdCSCs (Fig. 3C,D). The contribution of AXL to CSCs was also confirmed in Lu99 cells and H1703-AXL cells: treatment with siAXL-1 and siAXL-2 significantly reduced the proportions of tumour spheres to 61.1% and 21.2%, respectively, in Lu99 cells (Supplementary Fig. S4). Furthermore, exogenous *AXL* expression stimulated tumour sphere formation in H1703 cells, which express AXL at lower levels than H1299 cells (Supplementary Fig. S5). These results suggest that AXL protein stimulates *ALDH1A1* and *SNAI2 (Slug)* expression, and increases ALDH protein and ALDH activity, suggesting that AXL is related to tumour sphere formation and stemness.

### Enhanced tumourigenicity of AXL-high clones and absent tumourigenicity of AXL-low clones

**Figure 4:**
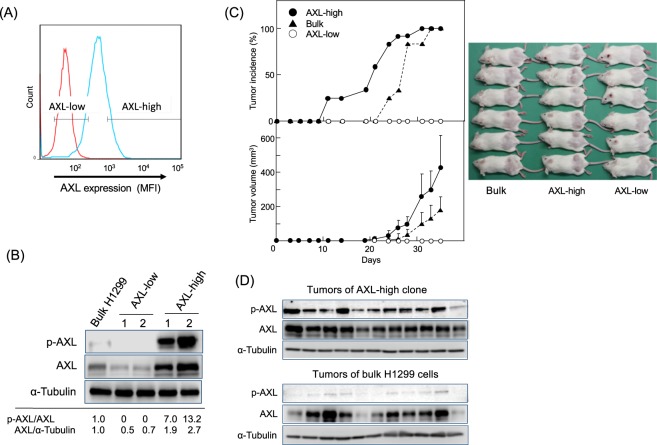
High tumourigenicity of AXL-high clone and no tumourigenicity of AXL-low clone. (**A**) AXL-high and AXL-low clones were isolated from 15% of the cells with the highest AXL expression and 15% of the cells with the lowest AXL expression, respectively, by flow cytometry using an anti-AXL antibody. (**B**) AXL and p-AXL levels in bulk H1299 cells, AXL-low, and AXL-high clones. Numbers indicate fold levels of p-AXL/AXL and AXL/α-tubulin compared with those of bulk H1299 cells. Full-length blots/gels are presented in Supplementary Information WB-3. (**C**) Tumour incidences and tumour volume in the AXL-high clone (•), bulk H1299 cells (▴), and the AXL-low clone (○). The same number of cells (1 × 10^6^) were subcutaneously transplanted into the backs of SCID/Beige mice. Tumour volume was calculated as described in the Materials and Methods. The image shows the mice in all three groups at 35 days after transplantation. (**D**) p-AXL and AXL levels in tumours of AXL-high clone and bulk H1299 cells. p-AXL, AXL, and α-tubulin (control) protein levels in tumours were examined by western blotting. Full-length blots/gels are presented in Supplementary Information WB-4. Tumour samples for western blotting were derived from the same experiment and gels/blots were generated in parallel and conducted three times.

To characterize the role of AXL in tumourigenicity, we sorted 15% of the H1299 cells as the AXL-high population and 15% as the AXL-low population by flow cytometry (Fig. 4A), and then established two cell clones: an AXL-high clone with high levels of both p-AXL and AXL, and an AXL-low clone with lower levels of both proteins. The p-AXL/AXL ratios were 7.0–13.2 and zero for the AXL-high and AXL-low clones, respectively, and the AXL/α-tubulin ratios were 1.9–2.7 and 0.5–0.7 for the AXL-high and AXL-low clones, respectively, compared with those of the bulk H1299 cells (Fig. 4B).

The same amounts (1 × 10^6^ cells) of the two clones and the bulk H1299 cells as a control were subcutaneously transplanted into SCID/Beige mice. The AXL-high clone produced larger tumours more rapidly than the bulk H1299 cells, whereas the AXL-low clone developed no tumours at 12 injection sites in six mice by day 35 of the experiment. Importantly, all the AXL-high clones had produced tumours by day 31 (Fig. 4C). Tumours derived from the AXL-high clone contained high levels of p-AXL and AXL compared with those from the H1299 cells (Fig. 4D). These results indicated that p-AXL and AXL proteins are important for high tumourigenicity.

### EGCG inhibited the tumour growth of H1299 cells by reducing p-AXL, ALDH1A1, and SLUG

**Figure 5:**
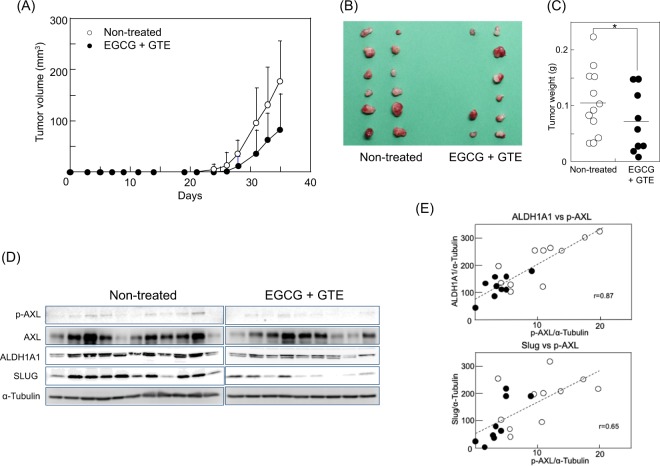
Oral administration of EGCG and GTE inhibited the tumour growth of H1299 cells by reducing p-AXL, ALDH1A1, and SLUG. (**A**) Tumour volume of the non-treated H1299 group (○) and the EGCG + GTE group (•). Mice in the EGCG + GTE group were given EGCG (100 mg/kg mice) via a gastric tube daily and 0.2% green tea extract as drinking water from one day before inoculating the cells. Tumour volume was calculated as described in the Materials and Methods. (**B**) The image shows all tumours that had developed in the two groups at 35 days post-transplantation. (**C**) Tumour weight of each tumour in the non-treated group (○) and in the EGCG + GTE group (•) *P < 0.05. (**D**) p-AXL, AXL, ALDH1A1, and SLUG protein levels in tumours were examined by western blotting. α-Tubulin was used as a control. Two membranes were simultaneously measured for intensity via LAS 4000 IR for p-AXL, ALDH1A1, and SLUG levels. The blots for AXL, p-AXL, and α-tubulin of the non-treated group are the same as those of the bulk H1299 cells group in Fig. 4D. Full-length blots/gels are presented in Supplementary Information WB-4. Tumour samples for western blotting were derived from the same experiment and gels/blots were generated in parallel and conducted three times. (**E**) Positive correlation between ALDH1A1 and p-AXL levels and between SLUG and p-AXL levels in tumours. Closed circles and open circles indicate those of each tumour in the EGCG-treated and non-treated groups, respectively. R values were 0.87 for ALDH1A1 vs. p-AXL and 0.65 for SLUG vs. p-AXL.

Since p-AXL is an essential factor for stemness, we examined the inhibitory effect of EGCG on tumour growth in H1299 cells *in vivo* using a mouse xenograft model. EGCG (100 mg/kg) and 0.2% GTE were administered from one day before injection of H1299 (10^6^ cells/injection) to the end of the experiment. The total amount of green tea catechins per day/mouse was estimated as 4.4 mg/mouse, the equivalent of 10 cups of green tea/day, which is the cancer preventive amount for humans^1–4^. The group treated with EGCG + GTE showed delayed tumour development (Fig. 5A); tumours were found in 75% (9/12) of the injected sites of the EGCG + GTE group, whereas tumours of the non-treated group developed in 100% (12/12) of the injected sites at 35 days after injection (Fig. 5B). The tumour size of the EGCG + GTE group was relatively smaller than that of the non-treated group (Fig. 5B), and the average tumour weight of the EGCG + GTE group was 0.05 ± 0.06 g, which was significantly lower than that of the non-treated group (0.10 ± 0.06 g; Fig. 5C). Notably, tumours of the EGCG + GTE group had lower levels of p-AXL and AXL compared with those of the non-treated group. In addition, EGCG + GTE inhibited AXL activation in tumours (Fig. 5D), which possessed lower levels of ALDH1A1 and SLUG proteins than those of the non-treated group (Fig. 5D). The p-AXL levels were well correlated with the ALDH1A1 and SLUG levels in the tumours, with r values of 0.87 and 0.65 (Fig. 5E). Thus, we concluded that the ALDH1A1 and SLUG axis regulated by AXL is involved in tumourigenicity and stemness, and that cancer prevention with EGCG mediates inhibition of the AXL/ALDH/SLUG axis.

## Discussion

Here we report for the first time the biophysical properties of H1299-sdCSC human lung cancer stem cells. AFM revealed that the average Young’s modulus value for H1299-sdCSCs was 1.52 kPa, which was 0.68-fold lower than that for the parental H1299 at 2.24 kPa. A previous investigation using AFM reported a 0.7-fold lower stiffness in a human hepatoma stem cell line, MHCC97H, compared with that of the parental cells^26^. Other researchers using a microfluidic device reported a higher deformability in ALDH^+^ CSCs isolated from an inflammatory breast cancer cell line, SUM149, than that in ALDH^−^ cells^27^. Cross *et al*. first reported that the average Young’s modulus value for metastatic cancer cells isolated from the pleural fluid of lung, breast, and pancreatic cancer patients tended to be approximately 0.33-fold lower than that of normal mesothelial cells^8^. We think that low stiffness, soft elasticity, high deformability, and metastatic potential are unique biophysical properties of CSCs isolated from various human cancers and that an investigation of the biophysical properties of CSCs is needed to advance cancer research. The cancer preventive activity of EGCG was effectively found in stiffened cells and in the tumour sphere formation of H1299-sdCSCs. Sun *et al*. reported that salinomycin, one of the most potent anti-CSC compounds screened by Weinburg *et al*., increases liver CSC stiffness in a manner similar to that of EGCG^28,29^.

OCT4, NANOG, and SOX2 are transcription factors that are required to maintain pluripotency^30^. Sphere-derived CSCs of colorectal cancer (HCT116-SDCSCs) express significantly higher levels of stemness marker genes, for example, 4.5-fold higher levels of *OCT4* and 3.2-fold higher levels of *NANOG* compared with those of the parental cells^25^. We also found that H1299-sdCSCs expressed *OCT4, NANOG*, and *SOX2* genes at levels of 5-fold more than the parental H1299 cells. Our study revealed that EGCG inhibited *ALDH1A1* and *SNAI2 (Slug)* expression and ALDH activity, but not *OCT4, NANOG*, or *SOX2* expression. Other investigators reported that EGCG inhibited the mRNA and protein expression of stemness markers, including OCT4 and NANOG, and increased the apoptotic markers BAX and caspase-8 in 20 human CSC lines collected from nine different cancer tissues^7^. But EGCG selectively inhibits ALDH and SLUG in this study. Some investigators reported that ALDH acts as a critical factor for cancer stemness^31^, and that SLUG promotes the expansion of CSCs in NSCLC by interacting with SOX9^32^. However, the mechanism of ALDH and SLUG has not yet been elucidated.

AXL is reported to induce EMT and self-renewal in breast cancer cells and chronic myelogenous leukemia CSCs^18,19^, and has a crucial role in the CSCs of various cancers. Kinome-wide shRNA screening revealed AXL to be an upregulated and phosphorylated receptor tyrosine kinase (RTK) amongst 82 RTKs in mesenchymal glioma stem-like cells^20^. Highly phosphorylated AXL is often found in NSCLC tissues and cell lines, but AXL is not expressed in normal lung tissues. Flow cytometry analysis revealed the presence of two populations with different AXL protein levels in H1299-sdCSCs, and the AXL-high and AXL-low clones showed different tumourigenicities. Importantly, AXL-high clones contained 3.5–6.1-fold higher p-AXL protein levels than AXL-low clones, and the AXL-low clone did not contain p-AXL, indicating the essential contribution of p-AXL to tumourigenicity. Our results are well supported by evidence that the p-AXL levels in tumours of the EGCG + GTE group were strongly reduced by 0.36-fold compared with those of the non-treated group, whereas AXL levels in tumours from both groups were nearly the same. These results clearly indicate that tumourigenicity and stemness require AXL activation with high p-AXL levels.

AXL activation is both GAS6-dependent and -independent^14^. Because AXL interacts with cMET, EGF receptor (EGFR), and human EGFR-related 2 (HER2), AXL–RTK crosstalk induces the transactivation of AXL in lung and breast cancers^16,33,34^. AXL expression was positively associated with GAS6 expression, and high coexpression of AXL/GAS6 was a strong risk factor in one study population^35^. EGCG treatment reduced AXL phosphorylation in the absence and presence of GAS6 (Supplementary Fig. S3A,B), indicating that EGCG inhibits AXL activation in both a GAS6-dependent and -independent manner. We previously reported that EGCG altered membrane organization to rigid stiffness, which we termed the “sealing effect of EGCG”^21^. Matsuzaki *et al*. recently found that treatment with the giant liposome of EGCG increased the stiffened bending rigidity of the lipid membrane by approximately 60 times more than that without EGCG^36^. Because altering the membrane organization with EGCG inhibits EGFR activation, EGCG likely inhibits AXL transactivation via AXL/RTK crosstalk.

AXL acts as a driver for acquiring chemoresistance against tyrosine kinase inhibitors (TKIs) and conventional anticancer drugs. Zhang *et al*. reported that activating AXL caused resistance to EGFR-targeted TKIs such as erlotinib in lung cancer, and that genetic and pharmacological inhibition of AXL restored TKI sensitivity^16^. Therefore, AXL inhibitors are attracting attention to overcome chemosensitivity, and clinical studies of several specific AXL inhibitors are underway^17^. EGCG reversed cisplatin resistance by downregulating AXL and TYRO3 expression in NSCLC and sensitizing 5-FU-resistant colorectal cancer^37^. These results suggest that the combined effects of EGCG and other compounds inhibit stemness through the AXL axis.

Protein tyrosine phosphatase 4 A (PTP4A) is a member of the phosphatase of regenerating liver (PRL) family, and PRLs are oncogenic across many human cancers^38^. Here, we discussed the results of AXL activation; however, PTP4A in human cancers should be considered in future studies.

In conclusion, AXL regulates the biophysical properties of CSCs, including low stiffness and soft elasticity. Stiffening CSCs via AXL inhibition is a critical mechanism of the multiple benefits provided by EGCG, including preventing cancer, inhibiting metastasis, and overcoming chemoresistance.

## Methods

### Cell cultures and reagents

The human NSCLC cell line, H1299 (ATCC CRL-5803), was purchased from American Type Culture Collection (ATCC; Manassas, VA, USA) and cultured in RPMI-1640 medium supplemented with 10% fetal bovine serum (Nichirei Bioscience Inc., Tokyo, Japan). The cells, which were confirmed as mycoplasma-free, were used at less than 20 passages and authenticated by short-tandem repeat profiling conducted by ATCC. EGCG of >98% purity was isolated from Japanese green tea leaves, and GTE was kindly provided by Dr. Atsuhi Takahashi at Saitama Tea Institute, as described previously^1,10^. Anti-AXL (R&D Systems, Minneapolis, MN, USA), anti-p-AXL (Y702) (Cell Signaling Technology, Inc., Danvers, MA, USA), anti-SLUG (Cell Signaling Technology, Inc.), anti-ALDH1A1 (Santa Cruz, Biotechnology, Inc., Santa Cruz, CA, USA), and anti-α-tubulin (Cell Signaling Technology, Inc.) antibodies were used for the experiments.

### Tumour sphere formation

H1299 cells were seeded at a density of 500 cells/well in serum-free medium (Dulbecco’s modified Eagle’s medium [DMEM]:F12 containing 0.45% methylcellulose, 50 ng/ml epidermal growth factor [EGF], 50 ng/ml fibroblast growth factor, and B27 supplement) in ultra-low-attachment 96-well plates (Corning Inc., Corning, NY, USA)^39^. Each treatment was conducted in six wells. After 3 weeks of culture, all wells were imaged using an All-in-One microscope (BZ-X700, Keyence, Tokyo, Japan), and the number of spheres measuring >100 µm on a minor axis were counted. Three independent experiments were conducted. Tumour spheres were separated from H1299-parental cells using a filter with a pore size of more than 77 µm (Spheroid Catch, Watson Co. Ltd, Tokyo, Japan) for gene expression analyses and AFM measurement.

### Cell stiffness measurement

Tumour spheres were separated using 0.1% trypsin-0.02% EDTA solution, then seeded into 6-cm dishes (Thermo Fisher Scientific, Cambridge, MA, USA) coated with biocompatible anchor for membrane (BAM; NOF Corporation, Tokyo, Japan)^23^. After 1.5 h, 16 force-distance curves per cell were obtained via force-map analysis using AFM (MFP-3D-Bio-J-AFM, Asylum Research, CA, USA). Using the Hertz model, Young’s moduli (*E*: kPa) were calculated from the force curves, as described previously^22^. Cell stiffness was obtained as the average Young’s modulus value of 30 cells from a lognormal fitting curve.

### Knockdown of *AXL* with siRNA

*AXL*-targeted siRNAs (s1845 and s1847, called siAXL-1 and siAXL-2, respectively) were purchased from Thermo Fisher Scientific. Supplementary Table S1 lists the siAXL sequences. A control siRNA (siControl) was obtained from OriGene Technologies, Inc. (Rockville, MD, USA). siRNA was transfected into H1299 cells using Lipofectamine RNAiMAX (Invitrogen, CA, USA) as described previously^11^. Cell stiffness, tumour sphere formation, and western blot analyses were conducted after 2 days.

### Western blot analysis

Cells or tumours were lysed in strong lysis buffer containing 20 mM Tris-HCl at pH 8.0, 150 mM sodium chloride, 1% Triton X-100, 0.1% sodium dodecyl sulfate (SDS), 1% sodium deoxycholate, 10 µg/ml aprotinin, 10 µg/ml leupeptin, 1 mM phenylmethylsulfonyl fluoride, 1 mM sodium orthovanadate, 0.5 mM sodium pyrophosphate, and 5 mM sodium fluoride, as described previously^11^. The lysates were subjected to SDS-PAGE, and then transferred onto a nitrocellulose membrane. The membrane was then incubated with primary antibody overnight at 8 °C, then incubated with the appropriate horseradish peroxidase-conjugated secondary antibody against rabbit IgG or mouse IgG. Specific bands were then detected using ImmunoStar LD (Wako Pure Chem. Ind. Ltd., Tokyo, Japan) using the C-DiGit Chemiluminescent Western Blot Scanner (LI-COR Bioscience Inc., Lincoln, NE, USA) or the LAS 4000 IR multi-color (Fujifilm Co, Tokyo, Japan). Band intensity was measured using C-DiGit or ImageJ (National Institute of Health, Bethesda, MD, USA). α-Tubulin was used as an internal control. At least three independent experiments were conducted.

### Quantitative real-time RT-PCR (qRT-PCR)

Total RNA isolated from the spheres was reverse transcribed into cDNA using oligo(dT)_16_ and MuLV reverse transcriptase (Thermo Fisher Scientific), and real-time PCR was conducted using SYBR Green I (LightCycler 480, Roche Lifescience, Basel, Switzerland) as described previously^40^. The primers used are indicated in Supplementary Table S2. *GAPDH* was used as an internal control, and relative gene expression was calculated as the fold expression compared with that of the control tumour spheres or H1299-parental cells. At least three independent experiments were conducted.

### Flow cytometry analysis of AXL

AXL protein level was examined via flow cytometry after incubation with anti-AXL antibody. Briefly, tumour spheres were treated with 0.1% trypsin and 0.02% EDTA solution containing 200 U/ml DNase I and 5 mM MgCl_2_ for 3 min at 37 °C. Separated sphere-derived cells were incubated with anti-AXL antibody for 30 min on ice, washed, and further incubated with AlexaFluor647-conjugated anti-goat IgG antibody (Invitrogen, Waltham, MA, USA) for 30 min on ice. Fluorescence intensity was determined via flow cytometry (BD FACSCanto II, BD Bioscience, San Jose, CA, USA)^40^. The experiments were independently conducted three times. Fifteen percent of both the AXL-high and AXL-low populations from H1299 cells were sorted using the BD FACS AriaIII (BD Bioscience) and cloned by seeding a single cell into a 96-well plate to obtain AXL-high and AXL-low clones.

### Aldefluor assay

ALDH activity was detected using the Aldefluor assay kit (StemCell Technologies, NC, USA) per the manufacturer’s instructions. Separated sphere-derived cells were incubated with BODIPY-aminoacetaldehyde (BAAA) for 30 min at 37 °C. Cells that catalysed BAAA to BODIPY-aminoacetate were analysed by flow cytometry (BD FACSCanto II, BD Bioscience) to determine ALDH-positive cells. The baseline of fluorescence was determined using the ALDH inhibitor, N,N-diethylaminobenzaldehyde (DEAB)^24^. The percentage of ALDH-positive cells was analysed using FlowJo software (FlowJo, LLC, Ashland, OR, USA). The results are expressed as the means of three independent experiments ± SD.

### Immunocytochemistry

Tumour spheres were fixed with 4% paraformaldehyde containing 0.2% Triton X-100 for 20 min and then incubated with 25% BlockAce (KAC Co. Ltd, Tokyo Japan) for 10 min. Anti-ALDH1A1 antibody (×500 dilution in BlockAce) was used as the first antibody and AF488-labeled anti-mouse IgG was used as the second antibody. Stained spheres were observed with a fluorescent microscope (BIOREVO BZ-9000, Keyence, Osaka, Japan). The average fluorescent intensity was obtained by BZ-X Analysis Application (Keyence).

### Tumour xenograft experiments

AXL-high or AXL-low clones (1 × 10^6^ cells/100 µl saline) were injected at two sites of the subcutaneous flanks of six 5-week-old female SCID/Beige mice. Tumour growth was monitored for 35 days by taking caliper measurements of the tumour size. Tumour volume was calculated by the formula, width^2^ × length/2^11^. EGCG (100 mg/kg) was orally administered using an intubation tube, and 0.2% GTE solution was additionally given as drinking water. EGCG + GTE was ingested from one day before injection of bulk H1299 cells until the end of the experiment. Tumour growth in the EGCG + GTE group was compared with that of the non-treated mice injected with bulk H1299 cells. All animal experiments were performed in accordance with the protocol approved by the International Animal Care and Use Committee of Saitama University (H30-A-1-12).

### Statistical analysis

Statistical analyses were performed using one-way analysis of variance followed by Dunnett’s test, while the Wilcoxon-Mann-Whitney test was used for analysing Young’s modulus and the animal experiments. Each experiment was conducted independently at least three times, and values are expressed as the mean ± SD. *P* < 0.05 was considered statistically significant.

## Supplementary information

Supplementary figures and tables.
