# Supplementary material for: (−)-Epigallocatechin gallate inhibits stemness and tumourigenicity stimulated by AXL receptor tyrosine kinase in human lung cancer cells
Source: Sci Rep. 2020 Feb 12;10:2444. doi: 10.1038/s41598-020-59281-z (PMC7016176; doi:10.1038/s41598-020-59281-z)
Supplement: Supplementary file 1 — Supplementary figures and tables. [file 41598_2020_59281_MOESM1_ESM.pdf]

(−)-Epigallocatechin gallate inhibits stemness and tumourigenicity stimulated by AXL receptor tyrosine kinase in human lung cancer cells

Kozue Namiki[1,2†], Pattama Wongsirisin[1,2†], Shota Yokoyama[1,2], Motoi Sato[1,2], Anchalee Rawangkan[1,2,3], Ryo Sakai[1,2], Keisuke Iida[1,2,4] & Masami Suganuma[1,2,*]

[1]Graduate School of Science and Engineering, Saitama University, Saitama 338-8570, Japan
[2]Research Institute for Clinical Oncology, Saitama Cancer Center, Saitama 362-0806, Japan
[3]School of Medical Science, University of Phayao, Phayao, Thailand 56000
[4]Molecular Chirality Research Center and Department of Chemistry, Graduate School of Science, Chiba University, Chiba 263-8522, Japan

*Correspondence to: masami0306@mail.saitama-u.ac.jp
†Co-first authors with equal contribution

Supplementary Fig. S1

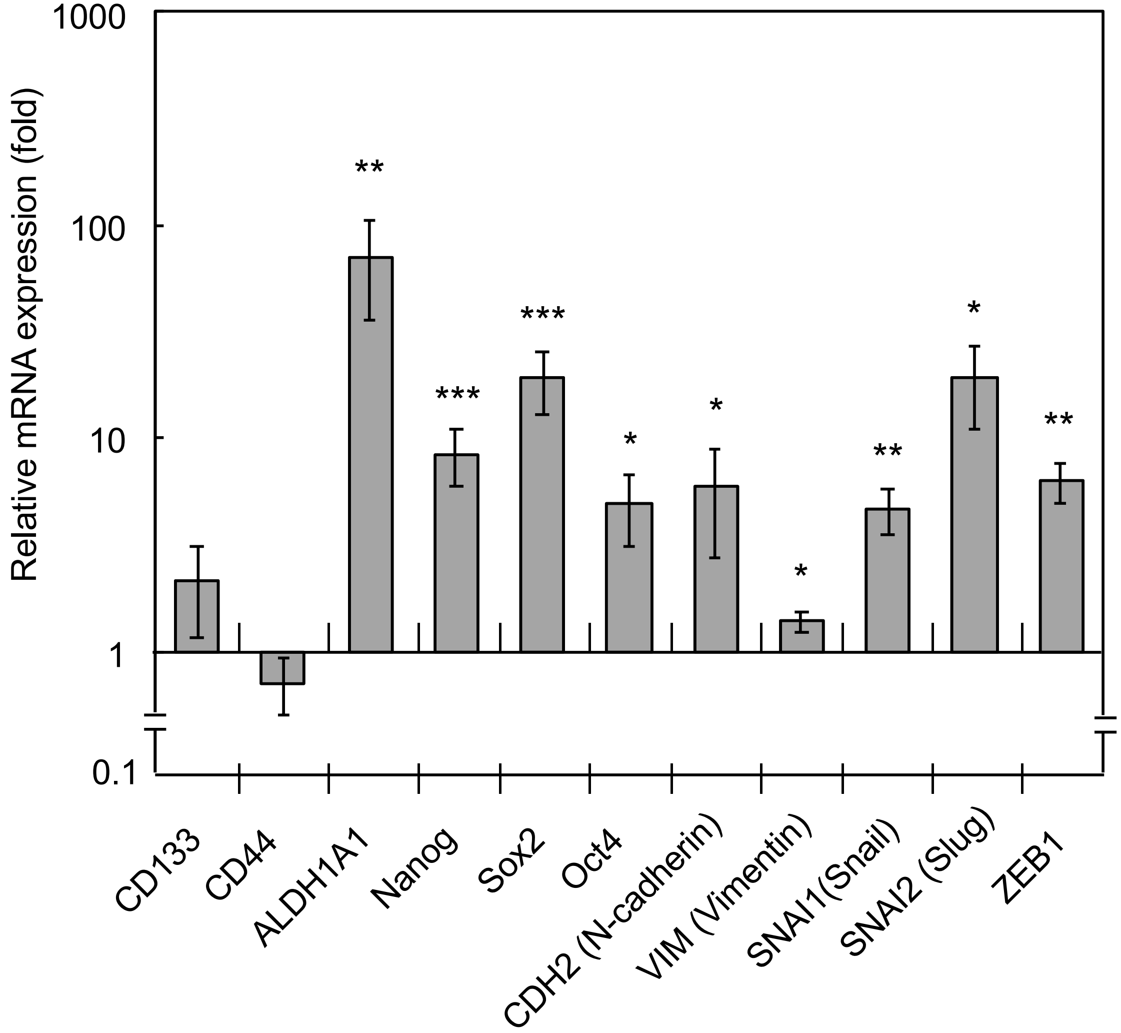

Expression of stemness-marker genes and EMT-related genes in H1299-sdCSCs.
Numbers indicate the relative mRNA expression compared with that in H1299-parental cells.
Results are mean ± SD from three independent experiments.
***: $p<0.001$, **: $p<0.01$, *: $p<0.05$

Supplementary Fig. S2

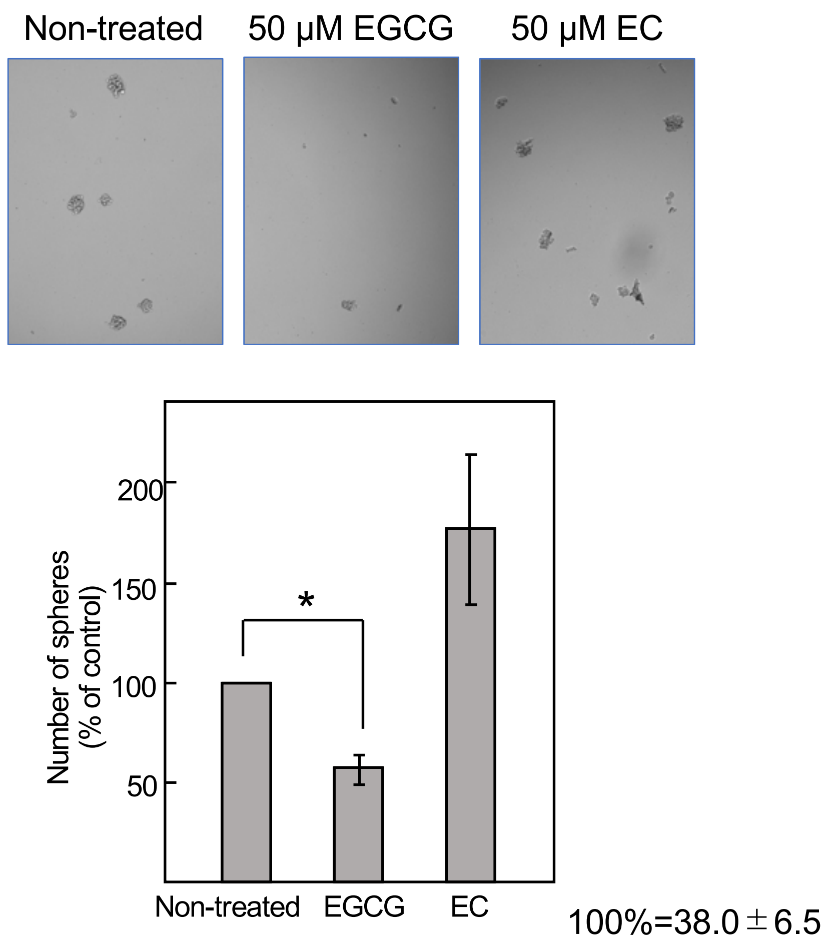

EGCG inhibited tumor sphere formation in Lu99 cells. Lu99 cells were cultured in serum-free medium in the presence of 50 µM EGCG or 50 µM EC for one week. (A) Percentage of tumor spheres of non-treated cells are shown in the graph. Photos show representative tumor spheres. The results are the mean ± SD of three independent experiments. *: $p<0.05$

Supplementary Fig. S3

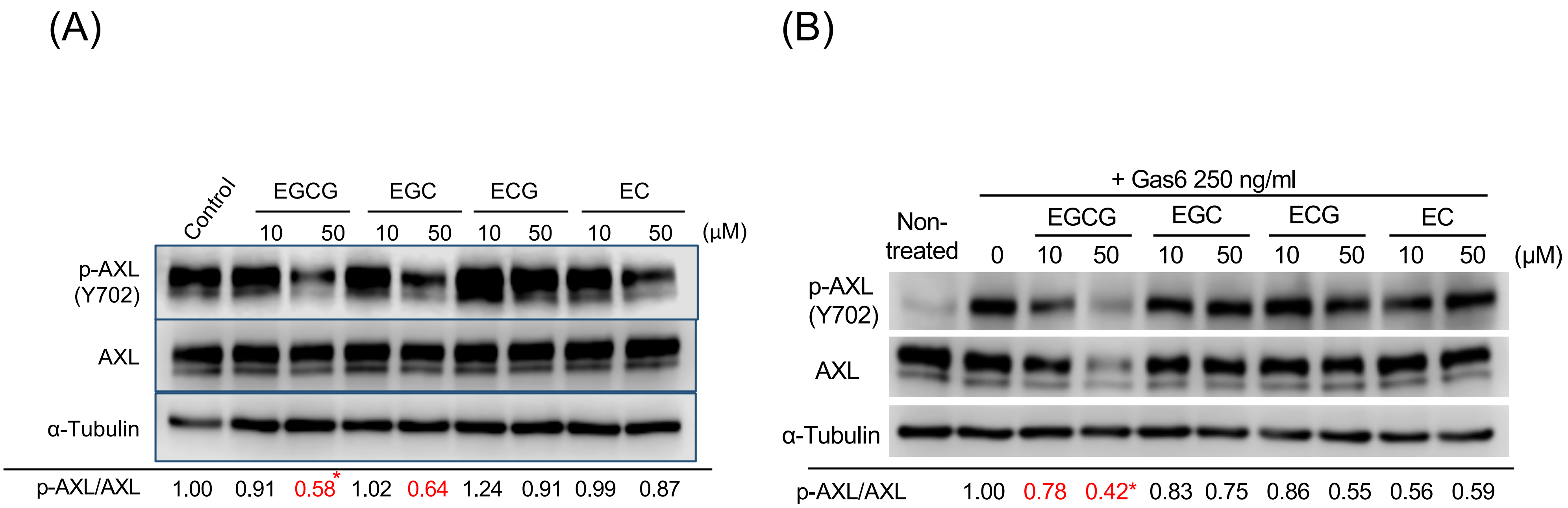

EGCG reduced the p-AXL levels compared with those of the H1299-parental control cells in the absence and presence of GAS6. α-Tubulin was used as a control. Numbers indicate relative p-AXL/AXL protein levels compared with those in the H1299-parental control cells. Full-length blots/gels are presented in Supplementary Information WB-1 and WB-5. Three independent experiments were conducted.

*: $p<0.05$

Supplementary Fig. S4

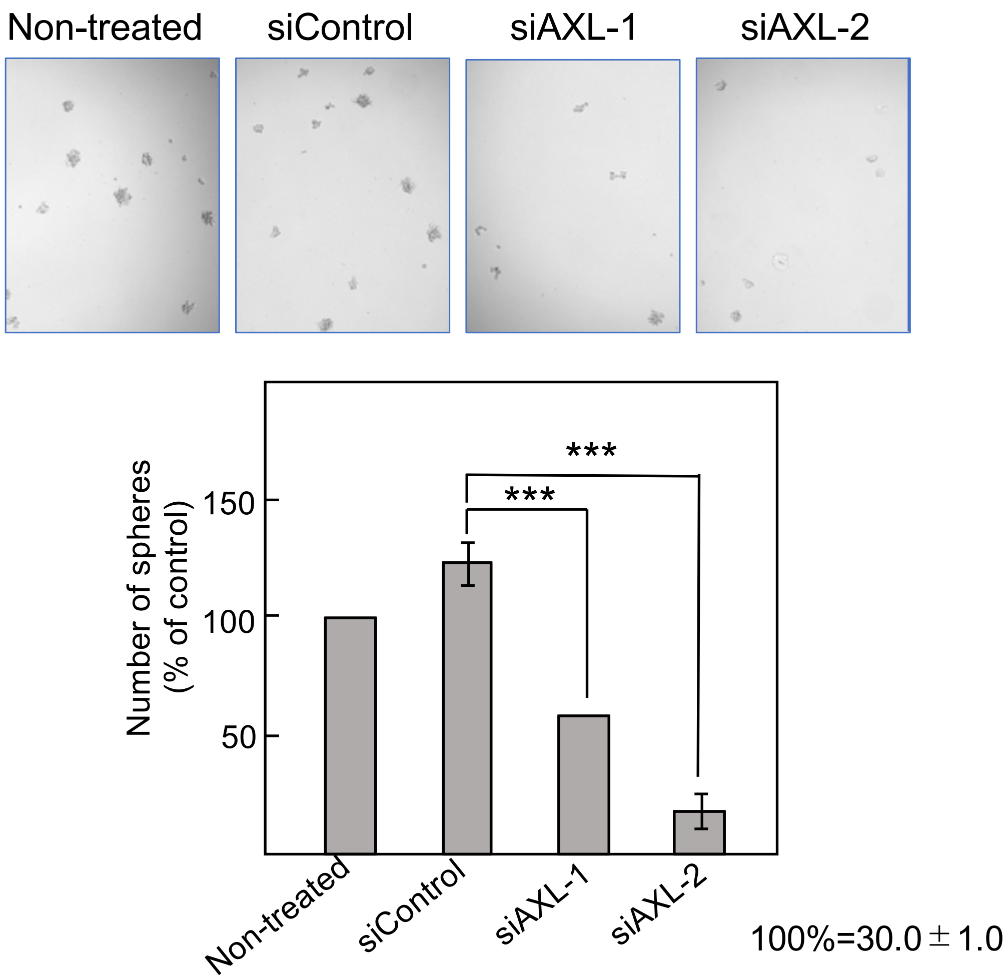

Knockdown of AXL with si AXL-1 and siAXL-2 inhibited tumor sphere formation in Lu99 cells. siAXLs and siControl were treated for 2 days, then the treated cells were cultured in serum-free medium for 1 weeks. (A) Photos show representative tumor spheres. Percentage of tumor spheres of non-treated cells are shown in the graph. The results are the mean ± SD of three independent experiments. (B) Reduction of ALDH1A1 protein in EGCG-treated tumor spheres in Lu99 cells.
***: $p<0.001$

Supplementary Fig. S5

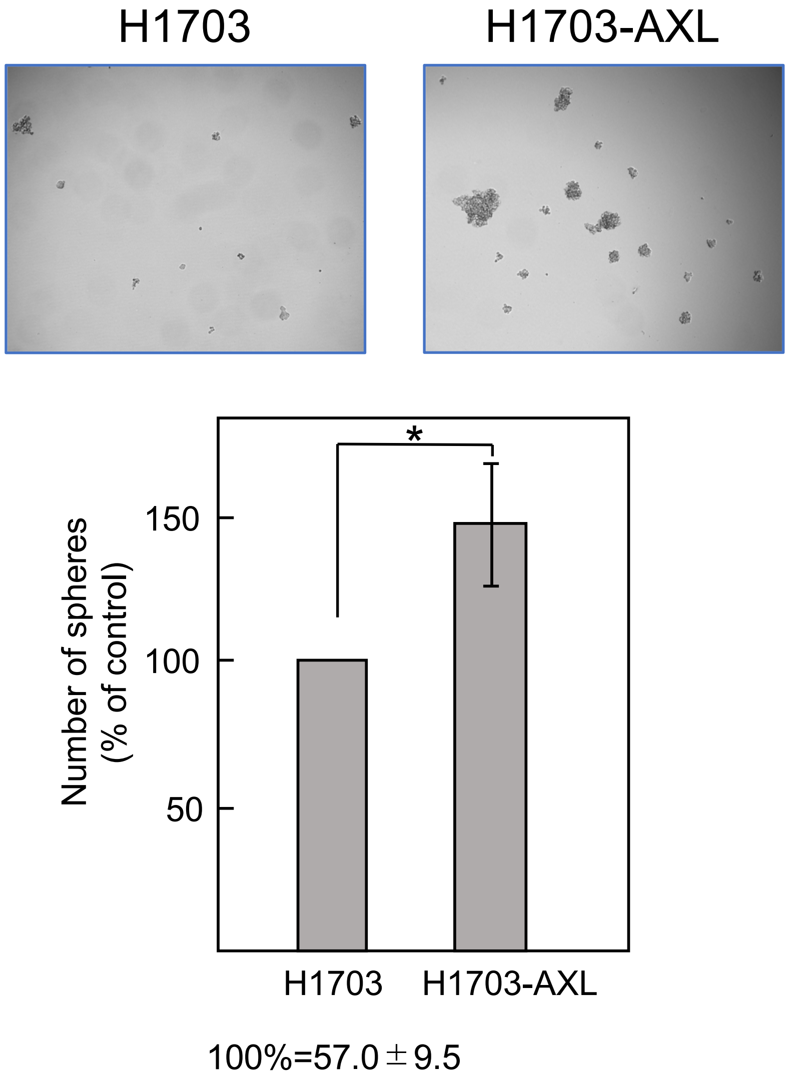

Exogenous *AXL* gene expression stimulates tumor sphere formation. H1703-AXL or H1703 cells were cultured in serum-free medium for 1 week. Photos show representative tumor sphere. Percentage of tumor spheres of non-treated cells are shown in the graph. The results are the mean ± SD of three independent experiments. *: $p<0.05$

Supplementary Table S1

siRNA	Sequence
siControl	—
siAXL-1	GGAACTGCATGCTGAATGA
siAXL-2	CAGCGAGAUUUAUGACUAU

Supplementary Table S2

Primer		Sequence (5' → 3')
GAPDH	Forward	TGGTATCGTGGAAGGACTCATGAC
	Reverse	ATGCCACTCAGCTTCCCGTTCAGC
AXL	Forward	GTGGGCAACCCAGGGAATATC
	Reverse	GTACTGTCCCGTGTGGGAAAG
Prominin-1 (CD133)	Forward	AGTCGGAAACTGGCAGATAGC
	Reverse	GGTAGTGTTGTACTGGGCCAAT
CD44	Forward	TCCAACACCTCCCAGTATGACA
	Reverse	GGCAGGTCTGTGACTGATGTACA
Nanog	Forward	TTTGTGGGCCTGAAGAAAACT
	Reverse	AGGGCTGTCCTGAATAAGCAG
Sox2	Forward	TACAGCATGTCCTACTCGCAG
	Reverse	GAGGAAGAGGTAACCACAGGG
POU5F1B (Oct4)	Forward	GTGTTCAGCCAAAAGACCATCT
	Reverse	GGCCTGCATGAGGGTTTCT
ALDH1A1	Forward	GCACGCCAGACTTACCTGTC
	Reverse	CCTCCTCAGTTGCAGGATTAAAG
CDH1 (E-cadherin)	Forward	CGACCCAACCCAAGAATCTA
	Reverse	AGGCTGTGCCTTCCTACAGA
CDH2 (N-cadherin)	Forward	CCTTGTGCTGATGTTTGTGG
	Reverse	TGGATGGGTCTTTCATCCAT
Vimentin	Forward	GGCTCAGATTCAGGAACAGC
	Reverse	TTCCAGGGACTCATTGGTTC
SNAI1 (Snail)	Forward	TCGGAAGCCTAACTACAGCGA
	Reverse	AGATGAGCATTGGCAGCGAG
SNAI2 (Slug)	Forward	TGTGACAAGGAATATGTGAGCC
	Reverse	TGAGCCCTCAGATTTGACCTG
ZEB1	Forward	TTACACCTTTGCATACAGAACCC
	Reverse	TTTACGATTACACCCAGACTGC
